# Anatomy and Histochemistry of the Vegetative System of *Brachystele guayanensis* (Lindl.) Schltr. (Orchidaceae), a Potential Medicinal Species

**DOI:** 10.3390/plants12142635

**Published:** 2023-07-13

**Authors:** Igor Soares dos Santos, Marcos José da Silva

**Affiliations:** 1 Post-Graduate Program in Plant Biology, Department of Botany, Institute of Biosciences, São Paulo State University (Unesp), Botucatu 18618-970, SP, Brazil; 2 Laboratory of Molecular Systematics and Plant Taxonomy, Department of Botany, Institute of Biological Science, Federal University of Goiás (UFG), Goiânia 74001-970, GO, Brazil; marcos_agrorural@hotmail.com

**Keywords:** alkaloids, ergastic substances, leaf, micromorphology, orchids, rhizomes, roots

## Abstract

The orchid genus *Brachystele* Schltr. (Orchidoideae, Cranichideae, Spiranthinae) comprises 20 species distributed from Mexico to Argentina, with 10 species found in Brazil. Anatomical studies of Orchidoideae Lindl. have been scarce, and the anatomy and histochemistry of *Brachystele* are still largely unknown. In this study, we conducted a characterization of the vegetative organs of *B. guayanensis* (Lindl.) Schltr. using standard anatomical and histochemical microtechniques. In this study, we provide the first information about the anatomy and histochemistry of *Brachystele*. The studied species was observed to display anatomical characters commonly found in the vegetative organs of representatives of the Cranichideae tribe (e.g., uniseriate epidermis; homogeneous mesophyll with 6–11 layers; rhizomes with rings of fibers; vascular bundles in the form of “^” or “v”; fleshy roots with uniseriate velamen, simple trichomes, and spiranthosomes). Others can be interpreted as adaptive strategies conditioned by the environment and their terrestrial life form (e.g., cuticle thickness; amphistomatic leaves; roots with reduced velamen compared to the cortex (18–20 layers); and raphides). In this study, cataphylls, and the presence of spiranthosomes in leaves, including stomatal guard cells, as well as alkaloids in these structures, are anatomically described for the first time in Orchidaceae. The presence of hyphae and pelotons in the stem of *B. guayanensis* is described for the first time in Cranichideae. Histochemical tests confirmed the presence of lignin, proteins, and alkaloids, the lipidic nature of the cuticle, starch grains stored in spiranthosomes, and the composition of the raphides. Alkaloids were observed in abundance, particularly in the roots, suggesting a potential role in defense against pathogens and herbivores, as well as potential medicinal activities, as seen in phylogenetically related groups to *Brachystele*.

## 1. Introduction

Orchidoideae Lindl. (Orchidaceae) is monophyletic and comprises approximately 3650 species and 208 genera, distributed throughout the temperate and tropical regions of the Old and New World [1,2]. In Brazil, there are approximately 363 species and 35 genera, with 205 species being endemic [3]. It is characterized by the presence of terrestrial species with an erect fertile anther, usually with sectile pollinae [4]. In addition to bringing together economically important species cultivated around the globe, especially for their potential as ornamental, food, and medicinal plants, particularly in the Old World [5,6,7], Orchidoideae are morphologically and anatomically diverse [1,2,8], presenting the species with interesting interactions with symbiotic microorganisms (e.g., fungi), as well as a range of adaptive strategies conditioned to the heterogeneous types of environments they occupy and to the interactions between biotic species, such as their pollinators, among others [1,8,9]. Additionally, they possess a wide range of biologically active substances [5,6,7].

In anatomical terms, Orchidoideae is poorly studied since most of the species sampled in previous studies [8,10] occur outside the neotropical region—one of its centers of diversity—where many of its species are endemic [1,2] and are subject to conditions and environmental factors that differ from other parts of the world. Although the phytochemistry of some species is relatively known, especially those occurring in the Old World [5,7,11], they are used in the treatment of various diseases because they have compounds with biological activities (e.g., anti-inflammatory, anti-Hepatitis B, neuroprotective, anti-tumor, antioxidant, among others), thus highlighting their potential as medicinal plants. Studies with this aspect are scarce in the neotropical region, especially in Brazil [12,13], as well as those directed to the histochemistry of the vegetative organs of their representatives [14,15,16], showing an important gap for the development of promising studies with different aspects in Orchidaceae in general.

Among the genus of Orchidoideae, *Brachystele* Schltr. (Cranichideae, Spiranthinae) comprises 20 species distributed from southern Mexico to northern Argentina [17]; 10 species occur in Brazil, of which three are endemic [3]. The taxon is terrestrial, herbaceous, with short rhizomes, fleshy roots, leaves arranged in rosettes, lateral racemes with small flowers, resupinate, usually indumented, with a bilobed stigma [3,17].

Anatomical descriptions of the vegetative organs of *Brachystele* are currently limited to the work of Bernal et al. [15], who examined the root trichomes of representatives of Spiranthinae Lindl. ex Meisn. as well as *B. widgrenii* (Rchb.f.) Schltr. However, there have been no histochemical studies of any *Brachystele* species, although some medicinal properties of *B. dilatata* (Lindl.) Schltr. and *B. unilateralis* (Poir.) Schltr. were mentioned (e.g., their diuretic and carminative property) in ethnobotanical studies [18,19].

**Figure 1:**
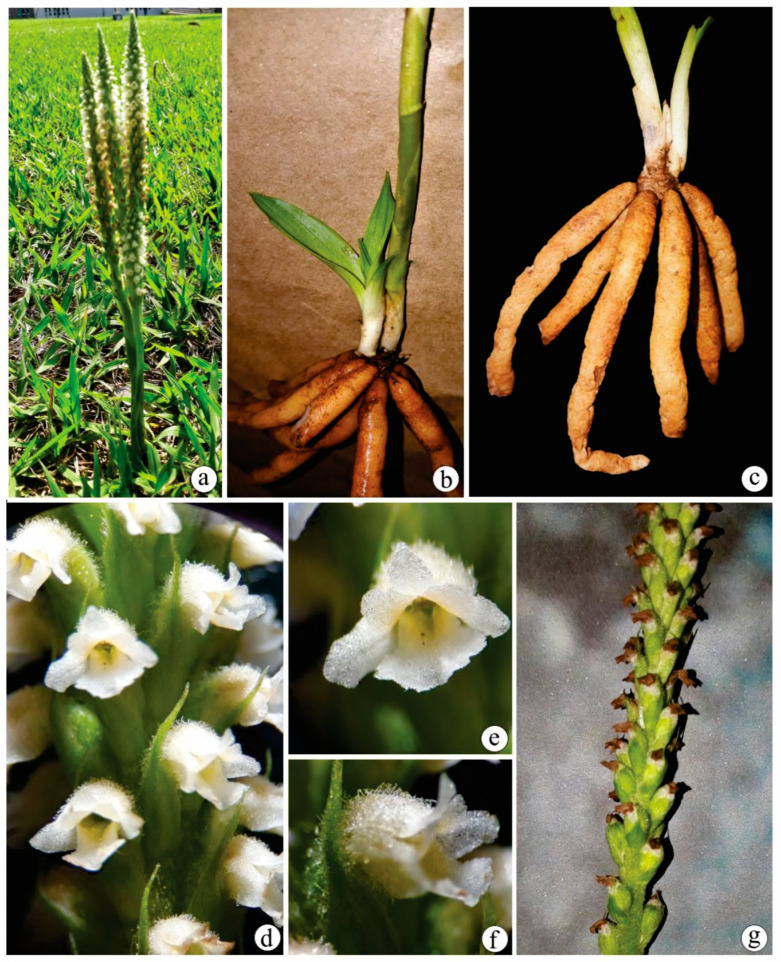
*Brachystele guayanensis* (Lindl.) Schltr. (**a**) habit; (**b**,**c**) details of leaf, short rhizome, tuberous roots, and base of the inflorescence axis; (**d**) detail of the inflorescence; (**e**) flower frontal view; (**f**) flower lateral view; (**g**) capsules. Photographs by Igor Soares dos Santos.

We provide here anatomical and histochemical characterizations of the vegetative organs of *B. guayanensis* (Lindl.) Schltr. (Figure 1).

## 2. Results and Discussion

### 2.1. Anatomical Data

#### 2.1.1. Leaf Anatomy

**Figure 2:**
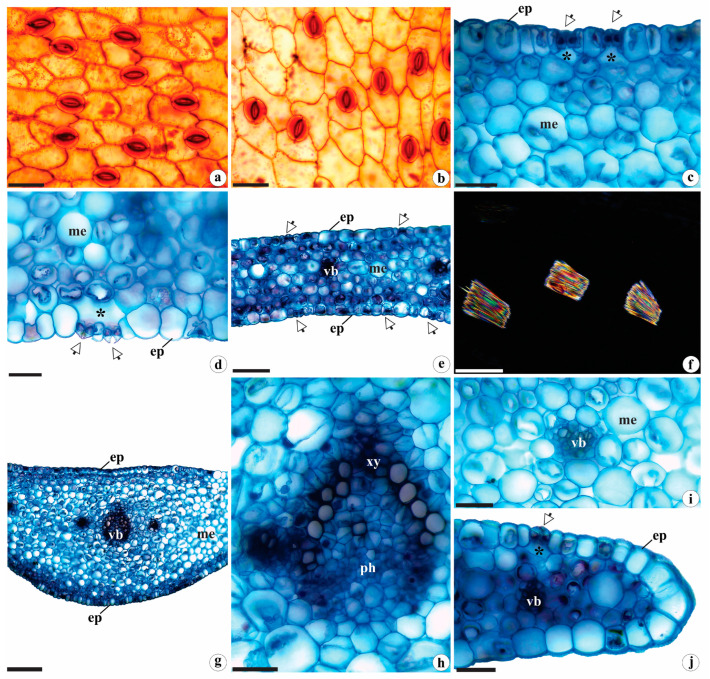
Leaf anatomy of *Brachystele guayanensis* (Lindl.) Schltr.; (**a**,**b**) epidermis in frontal view; (**c**–**j**) cross-sections; (**a**,**c**) detail of the adaxial surface; (**b**,**d**) detail of the abaxial surface; (**e**) leaf blade; (**f**) detail of raphides under polarized light; (**g**) midrib; (**h**) detail of the vascular bundle of the midrib; (**i**) detail of the smaller-caliber vascular bundle; (**j**) margin. ep = epidermis; me = mesophyll; ph = phloem; vb = vascular bundle; xy = xylem; * = substomatic chamber; arrows = stomata. Scales: (**a**–**d**,**f**,**h**–**j**) = 50 µm; (**e**,**g**) = 200 µm. Photomicrographs by Igor Soares dos Santos.

In frontal view, the leaf epidermis of *B. guayanensis* is covered by a striated cuticle (Figure 2a,b). It is composed of polygonal cells with both straight and curved walls (Figure 2a,b)—characters that have also been reported for *Microchilus arietinus* (Rchb. f. and Warm.) Ormerod and *Zeuxine strateumatica* (L.) Schltr. by Andreota et al. [20] and Bona et al. [16] and described for *Aa paleacea* (Kunth) Rchb. f. and *Pterichis multiflora* (Lindl.) Schltr. by Corredor and Arias [21]. Anomocytic stomata were observed on both faces of the leaf blade of *B. guayanensis* (Figure 2a,b), similar to other species in the Orchidaceae family [8,22,23].

In cross-section, the epidermis of *B. guayanensis* is covered by a thin, striated cuticle. It is uniseriate and composed of rounded, oblong cells with slightly thickened external periclinal walls (Figure 2c,d)—aspects reported for other members of tribes Diurideae Endl. [24], Orchideae Small [25,26,27], and Cranichideae [16,20,21]. The leaves are amphistomatic (Figure 2c–e), with stomata at the same level as common cells of the epidermis. The substomatal chambers are wider than the suprastomatal chambers (Figure 2c,d)—characteristics related to reducing water losses and evapotranspiration [28,29]. Amphistomatic leaves are commonly observed in plants that have both high photosynthetic capacities and high stomatal conductance, especially those that grow in open and sunny environments [30,31], as studied here.

The mesophyll of *B. guayanensis* is homogeneous and consists of 6–11 layers of rounded cells with varying dimensions (Figure 2e). These cells are interspersed with crystalliferous idioblasts containing raphides (Figure 2f)—a pattern cited for different groups of Orchidaceae [8,10,23]. Collateral vascular bundles surrounded by a parenchyma sheath were observed in the median portion of the mesophyll (Figure 2e). The bundle corresponding to the midrib is flat-convex (Figure 2g) and has the largest caliber; the elements of that vessel are arranged in a “^” (Figure 2h). Smaller bundles (Figure 2i) with smaller calibers are interspersed. This same pattern has been reported in other taxa by the authors cited above. The leaf margins of *B. guayanensis* are straight and rounded (Figure 2j).

#### 2.1.2. Rhizome Anatomy

**Figure 3:**
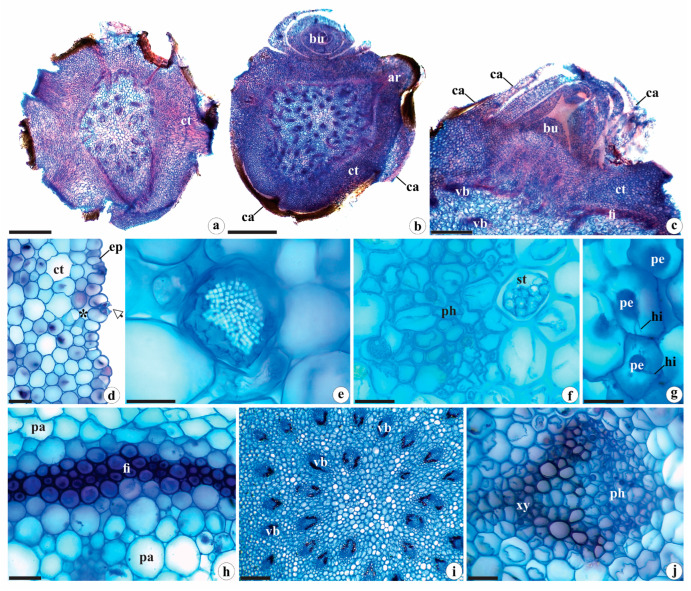
Rhizome anatomy of *Brachystele guayanensis* (Lindl.) Schltr.; (**a**,**b**) general aspect; (**c**) detail of the buds with leaf primordia and cataphylls; (**d**) detail of the epidermis and portion of the cortical parenchyma; (**e**) raphides; (**f**) starch grains gathered in spiranthosomes; (**g**) detail of hyphae and pelotons; (**h**) detail of fiber ring; (**i**) detail of the central portion of the rhizomes and arrangement of the vascular bundles; (**j**) detail of the vascular bundle. ar = adventitious root; bu = buds; ca = cataphylls; ct = cortex; ep = epidermis; fi = fibers; vb = vascular bundles; hi = hyphae; pa = parenchyma; pe = pelotons; ph = phloem; st = starch grains gathered in spiranthosomes; xy = xylem; * = substomatic chamber; arrows = stomata. Scales: (**a**,**b**) = 1000 µm; (**c**) = 500 µm; (**i**) = 200 µm; (**d**,**f**–**h**,**j**) = 50 µm; (**e**) = 20 µm. Photomicrographs by Igor Soares dos Santos.

The anatomy of the rhizomes of *B. guayanensis* was observed to be similar to other representatives of Orchidoideae [8,21,23] in having rounded outlines and variable calibers (Figure 3a,b), with cataphylls in nodal regions or protecting the buds (Figure 3b,c). Cataphylls, whose anatomy is first described for Orchidaceae, have a uniseriate epidermis composed of quadrangular cells covered by a thin cuticle, and stomata facing the external portion (Figure 3b,c). The mesophyll is homogeneous, consisting of 6–8 layers, and contains collateral vascular bundles in the form of “^” (Figure 3b,c). The epidermis of the rhizomes is covered by a smooth, thin cuticle, and is uniseriate. It is composed of oblong, or occasionally, rounded common cells with thin cell walls (Figure 3d). Stomata were only observed on the exposed portions of the rhizome and were arranged at the same level as the other common cells, with only tiny substomatal and suprastomatal chambers (Figure 3d).

The cortex of *B. guayanensis* is surrounded by a fiber ring (Figure 3a,b,h) and consists of 16–22 layers of rounded parenchyma cells of varying sizes (Figure 3a,b) with small triangular intercellular spaces. Some of the parenchyma cells contain raphides (Figure 3e) and tiny starch grains enveloped by two limiting membranes and gathered in spiranthosomes (Figure 3f)—spherical specialized amyloplasts found in Cranichideae and interpreted as a synapomorphy of the tribe and are associated with nutrients storage [16,20,32]. While it is not entirely clear, some authors [33,34] attribute the polyhedral form of spiranthosomes to intracellular density. However, others believe that such structures reflect shared genetic factors and phylogenetic characters [35]. Furthermore, we believe that the presence of membranes surrounding the tiny starch grains possibly regulates the hydrolysis process, as well as the availability of sugars for the symbiotic fungi.

The arrangement of the fundamental tissue is similar to that described by Stern [8] and by Stern and Judd [36,37] for the aerial stems of other Orchidaceae groups (such as Vanilloideae [36,37]; Orchidoideae, tribes Diseae Dressler and Orchideae Small; and Epidendroideae Lindl., tribes Sobralieae Pfitzer and Triphoreae Dressler [8,38]), as well as other groups of Monocotyledons [39]. Although the endodermis (with Casparian strips) and/or pericycle are not very distinct, unlike other Cranichideae [8,16] as described here (Figure 3b), they may participate in the genesis of adventitious roots and therefore exhibit meristematic activity [39].

Fungal hyphae and pelotons were observed to be concentrated mainly in the more peripheral portions of the cortical parenchyma (Figure 3g), which aligns with the findings of Pereira et al. [40] for the roots of *Bulbophyllum* sp., *Campylocentrum organense* (Rchb.f.) Rolfe and *Gomesa crispa* (Lindl.) Klotzsch ex Rchb. f. According to these authors, the strategic positions of these structures contribute to the maintenance and (re)colonization of internal tissues, serving as important sources of inoculum for adventitious roots extending from the rhizome, corroborating the findings of Pridgeon [41] for terrestrial orchids of Orchidoideae (tribe Diurideae), and of Bougoure et al. [42], Uma et al. [43] and Suetsugu et al. [44] for Epidendroideae (*Eulophia epidendraea* C.E.C. Fisch., *Malaxis acuminata* D. Don, *Oreorchis indica* (Lindl.) Hook. f. and *Rhizanthella gardneri* R.S. Rogers). Such structures were also found in the rhizomes of epiphytic and rupicolous orchids of the Epidendroideae (*Promenaea rollisonii* (Rchb. f.) Lindl. and *P. xanthina* Lindl.) by Pedroso-de-Moraes et al. [45]. In this study, the presence of hyphae and pelotons in the stem of *B. guayanensis* is first reported for Cranichideae. According to some authors [46,47,48], the presence of mycorrhizae can act as extensions of the root system, maximizing the absorption and translocation of nutrients by plants (e.g., phosphorus, potassium, phosphate, manganese, and nitrogen), including copper and zinc, in addition to other immobile nutrients present in the soil [49,50,51]. Moreover, fungi improve structure, stability, and water retention in the soil [52]; increase tolerance to abiotic stresses [46], and contribute to the absorption of amino acids, ammonia, and nitrate from the rhizosphere, making these elements available in inorganic form to the host plant [48].

Between 28 and 45 scattered collateral vascular bundles can be observed internally, surrounded by a parenchyma sheath (Figure 3i), which is typical of Monocots [30,53,54]. These bundles are observed in the central portion of the organ analyzed (Figure 3a,b,i), and have been observed in other groups of the Orchidaceae family [8,36,37]. The vessel elements of the xylem are arranged in “V” formations within the collateral vascular bundles (Figure 3j), consistent with the findings of Andreota [55] for the tribe Cranichideae.

#### 2.1.3. Root Anatomy

**Figure 4:**
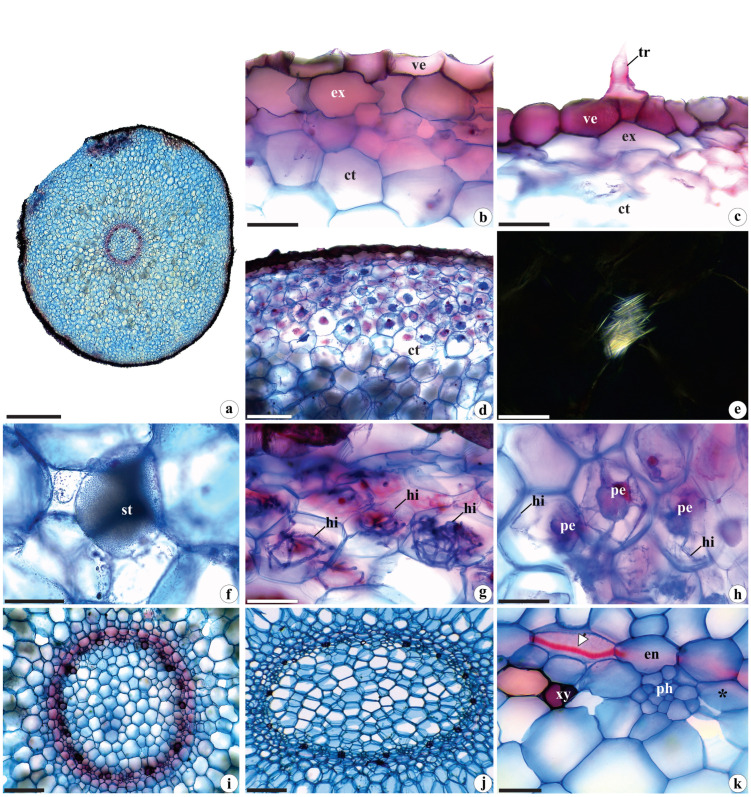
Root anatomy of *Brachystele guayanensis* (Lindl.) Schltr.; (**a**–**k**) cross-sections; (**a**) general aspect; (**b**,**c**) detail of the velamen, exodermis and trichomes; (**d**) detail of the cortex. Note the presence of hyphae and pelotons in more peripheral portions; (**e**) raphides under polarized light; (**f**) starch grains gathered in spiranthosomes; (**g**,**h**) detail of hyphae and pelotons in the cortical parenchyma; (**i**,**j**) vascular cylinder; (**k**) detail of the endodermis, pericycle, xylem and phloem. ct = cortex; en = endodermis; ex = exodermis; hi = hyphae; pe = pelotons; ph = phloem; st = starch grains gathered in spiranthosomes; tr = trichome; ve = velamen; xy = xylem; * = pericycle; white arrows = Casparian strips. Scales: (**a**) = 1000 µm; (**b**,**c**,**e**,**g**,**h**,**k**) = 50 µm; (**f**) = 20 µm; (**d**,**i**,**j**) = 200 µm. Photomicrographs by Igor Soares dos Santos.

*Brachystele guayanensis* has transversely circular roots of variable calibers (Figure 4a), which are anatomically similar to those found in other taxa of Cranichideae tribe [20,21,56]. These roots possess a uniseriate velamen of the “Spiranthes type”. The component cells are irregular, elliptic, and thin, with thin cell walls with helicoidal thickenings and small pores in the non-thickened regions (Figure 4b,c). Our observations align with those of Porembski and Barthlott [57] for *Pelexia dolichorhiza* Schltr. (=*Pachygenium pteryganthum* (Rchb. f. and Warm.) Szlach., R. González and Rutk.), *Prescottia colorans* Lindl. (=*P. stachyoides* (Sw.) Lindl.), and *Sauroglossum elatum* Lindl. Moreira and Isaias [58] reported that terrestrial orchids generally have thinner velamen compared to epiphytic or rupicolous species in terms of their numbers of layers. Moreira and Isaias [58], Pridgeon [59], and Chomicki et al. [60] noted that velamen serves multiple functions, such as protecting the roots from high solar radiation levels and excessive evapotranspiration losses, preventing overheating, and providing mechanical support to internal tissues, among others.

Simple, unicellular trichomes, such as those observed here (Figure 4c), were reported for: *Brachystele widgrenii*, *Lankesterella caespitosa* (Lindl.) Hoehne, *L. ceracifolia* (Barb. Rodr.) Mansf., *Pelexia orthosepala* (Rchb.f. and Warm.) Schltr, and *Sacoila lanceolata* (Aubl.) Garay. by Bernal et al. [15]; for *Cranichis candida* (Barb. Rodr.) Cogn. by Andreota et al. [20]; and for 11 species of *Aspidogyne* Garay and *Microchilus* C. Presl. by Bona et al. [16]. According to Stern et al. [8], Andreota et al. [20], and Bernal et al. [15], simple trichomes provide better attachment to the substrate and increase contact with that surface, thereby facilitating the absorption of water and mineral salts.

The root cortex consists of 18–20 layers of rounded parenchyma cells of varying sizes with innumerable small, triangular intercellular spaces (Figure 4d). In addition, the presence of hyphae and pelotons is observed, primarily concentrated in the more peripheral regions and underlying the exodermis (Figure 4d,g,h). Similar patterns have been reported in the roots of other Orchidaceae species by Pereira et al. [40]. The presence of symbiotic microorganisms in these roots is crucial for orchid seed germination, as they increase the surface area of the roots, thereby facilitating water and nutrient absorption [50,61]. For more detailed information, please refer to Section 2.1.2.

These characters are frequently observed in Orchidoideae [8,21,56]. In terrestrial orchids, including the species studied here, the roots are typically thick and fleshy, with a less expressive velamen and more pronounced cortex in terms of the number of layers. It is responsible for holding reserves of water, starch grains, and other nutrients. Within the cortical parenchyma, some cells contain raphides (Figure 4e), a feature observed in various groups of Monocotyledons, including Orchidaceae [53]. Additionally, polyhedral starch grains are found in spiranthosomes (Figure 4f), which are typical amyloplasts in Cranichideae [16,20,32].

The exodermis (Figure 4b,c), endodermis, and pericycle (Figure 4k) of *B. guayanensis* are uniseriate and composed of rounded, elliptical cells with thin walls. The cells of the exodermis are slightly thickened (Figure 4b,c) and the endodermis cells have evident Casparian strips (Figure 4k). Similar patterns can be observed in other terrestrial orchids, particularly in the subfamily Orchidoideae, such as in Orchideae [27] and Cranichideae [16,20,21].

The vascular cylinders in the roots of *B. guayanensis* exhibit variable calibers and can have circular or elliptic shapes. They also possess 12–14 protoxylem poles (Figure 4i,j), categorizing these roots as polyarchs, a common feature in Monocotyledons [30,54]. The xylem and phloem are interspersed within the vascular cylinder (Figure 4i–k). The central portion of the vascular cylinder is composed of parenchyma cells of varying sizes and shapes with tiny triangular intercellular spaces (Figure 4i,j), similar to those observed in other groups of Orchidaceae [8,23,56].

### 2.2. Histochemistry Data

**Figure 5:**
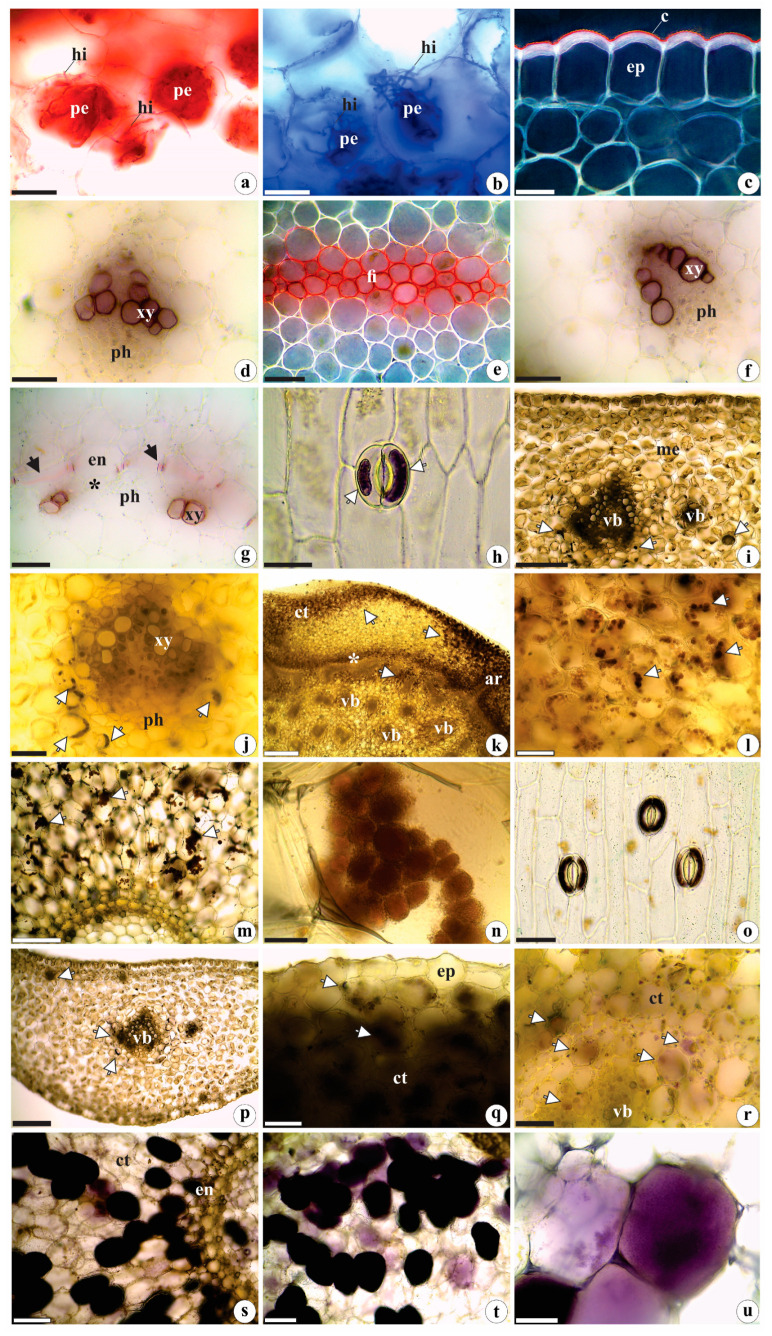
Histochemical tests in the vegetative organs of *Brachystele guayanensis* (Lindl.) Schltr.; (**a**–**g**,**i**–**n**,**p**–**u**) cross-sections; (**h**,**o**) epidermis in frontal view; (**a**,**b**,**g**,**m**,**n**,**s**–**u**) root; (**c**,**d**,**h**–**j**,**o**,**p**) leaf; (**e**,**f**,**k**,**l**,**q**,**r**) rhizome; (**a**,**b**) protein nature of fungal hyphae and pelotons; (**c**) detail of the cuticle (total lipids) under polarized light; (**d**–**g**) lignin (polarized light in (**e**)); (**h**–**n**) starch grains. Note the spiranthosomes in (**n**); (**o**–**u**) total alkaloids. c = cuticle; ct = cortex; en = endodermis; ep = epidermis; fi = fibers; hi = hyphae; me = mesophyll; pe = pelotons; ph = phloem; vb = vascular bundle; xy = xylem; * = fiber rings; black arrows = Casparian strips; white arrows = starch grains and alkaloids. Scales: (**a**,**b**,**d**–**h**,**j**,**l**,**o**,**q**–**t**) = 50 µm; (**c**,**n**,**u**) = 20 µm; (**i**,**m**,**p**) = 200 µm; (**k**) = 500 µm. Photomicrographs by Igor Soares dos Santos.

Coomassie blue and xylidine ponceau strongly stained the pelotons and hyphae, indicating their protein contents (Figure 5a,b). Sudan IV staining confirmed the lipidic nature of the waxy cuticle (Figure 5c), and the raphides were composed of calcium oxalate. Lignin was identified in the walls of xylem vessel elements (Figure 5d,f,g) and fiber rings (Figure 5e), as well as in Casparian strips (Figure 5g). Starch grains and alkaloids were identified within the stomata guard cells (Figure 5h,o), epidermal cells, mesophyll cells (especially in the vicinity of vascular bundles) (Figure 5i,j,p), the cortical parenchyma of rhizomes (and occasionally in the central portion) (Figure 5k,l,q,r), as well as in roots (where they were most abundant) (Figure 5m,n,s–u). The histochemical tests were negative for reducing sugars, phenolic compounds, and tannins.

The waxy cuticle performs several functions, including protection against solar radiation, overheating, and water loss from internal tissues. It also provides protection against the entry and attacks of pathogens and herbivores [62,63,64].

Calcium oxalate raphides, found in different groups of Orchidaceae and other Angiosperms [8,65], serve as defenses against herbivores, act in osmoregulation and in other metabolic activities requiring calcium, and have a role in the detoxification of aluminum, which is abundant in savanna soils, such as the red latosol (Oxisol) where the studied species grew [65,66].

Lignin, commonly deposited in vessel elements, fiber rings, and the Casparian strip of the vegetative organs of the studied species, confers stability, rigidity, and mechanical support to the cell walls and internal tissues [30,54]. The Casparian strip, along with suberin deposits (both hydrophobic substances), contribute to solute selectivity in the root endodermis and act as a barrier against apoplast movement, preventing the influx of ions from the vascular cylinder to the cortical region [67,68,69].

Starch grains, observed in spiranthosomes for the first time in leaves, were also found inside stomata guard cells of the studied species (Figure 5h). Starch grains in guard cells have been reported in other orchids [70] and other Angiosperms [71,72,73]. According to Appezzato-da-Glória and Carmello-Guerreiro [54], potassium levels in those structures appear to be associated with starch hydrolysis, which provides those organic anions. There is also evidence that malate, the regulator responsible for guard cell movements, can be synthesized through starch degradation [74,75].

According to Sut et al. [11] and Bulpitt et al. [76], many of the secondary metabolic substances identified in representatives of Orchidaceae (such as flavonoids, alkaloids, terpenes, glycosides) act in plant defenses against herbivores and pathogens and have bioactive pharmacological effects [77,78]. We believe that the presence of alkaloids in the stomata guard cells (first reported for orchid guard cells), and in the cortical parenchyma of rhizomes and roots, along with the identified raphides in *B. guayanensis,* suggests their defensive role against pathogens and herbivores, as mentioned by Franceschi and Nakata [65] and Vizzotto et al. [79] for plants in general. Similar to what has been postulated by Li et al. [80] for the vegetative organs of other orchids, the abundant presence of raphides and secondary metabolites (e.g., alkaloids) in *B. guayanensis,* especially in the reserve organs (e.g., the roots and rhizomes), are responsible for renewing their aerial portions and help prevent pathogens and herbivores from reaching the vascular system (which is usually found in more internal regions) and causing local and/or systemic damage.

Among the bioactive substances found in Orchidaceae species are alkaloids (e.g., dendrobin, nobilonin, dendroxin) and nitrogenous heterocyclic organic molecules derived from the secondary metabolism of amino acids (e.g., phenylalanine, lysine, arginine, tyrosine, tryptophan). More than 100 types of bioactive alkaloids have been identified in over 2000 orchid species [5,81]. These alkaloids have been found to be effective in treating gastrointestinal disorders and cardiovascular diseases and have shown anti-inflammatory, diuretic, analgesic, antioxidant, immunomodulatory, antipyretic, and antitumor activities [82,83,84]. The presence of alkaloids in *B. guayanensis* indicates its potential as a medicinal species. Further (bio)phytochemical investigations could be informative, including the subsequent isolation and toxicity testing of any bioactive substances and the characterization of their chemical nature.

## 3. Materials and Methods

For the anatomical studies, samples of vegetative organs (e.g., the mid-portions of the leaf blades, rhizomes, and roots) from five adult individuals of *B. guayanensis* (Figure 1) were collected in open fields near Bosque Auguste Saint-Hilaire, at Campus II (Samambaia) of the Federal University of Goiás (UFG), Goiânia, GO, Brazil. Botanical material collections followed the recommendations of Mori et al. [85], and voucher specimens were deposited in the UFG herbarium (registration numbers: *I.S. Santos 1160* and *1161*).

The collected samples for anatomical studies were fixed in 70% FAA (glacial acetic acid, formaldehyde, and 70% ethyl alcohol, 1:1:18) in hermetically sealed containers for 48 h. After this period, they were preserved in 70% ethyl alcohol [86]. For the anatomical descriptions, cross-sections were cut using a razor blade and clarified in a 20% aqueous solution of sodium hypochlorite (NaClO) (*v*/*v*) [87]. They were then stained with astra blue and safranin (9:1) [88] and mounted in aqueous glycerol solution (1:1). To analyze the leaf surface in frontal view, the epidermis was dissociated using the Jeffrey method [86]. For the procedures mentioned above, the slides were sealed with a colorless sealant and subsequently photomicrographed using a Leica ICC50 HD^®^ digital camera attached to a Leica DM500^®^ microscope, using Motic 2.0 Image Plus Software.

For histochemical studies, in natura samples obtained at the time of collection were stained with the following reagents: Coomassie blue and xylidine ponceau [89,90] for detecting proteins; ferric chloride for detecting phenolic compounds; acidified phloroglucinol for lignin; Sudan IV for total lipids [86]; Dittmar for alkaloids [91]; Fehling for reducing sugars [87]; Lugol for starch [92]; and hydrochloric vanillin to detect tannins [93]. To verify the chemical constitution of the crystals, 10% hydrochloric acid was used, following Chamberlain [94]. The descriptions of the examined organs were based on the terminologies used in the specialized literature [16,20,21].

## 4. Conclusions

In this study, we have provided the first information regarding the anatomy and histochemistry of *Brachystele*. The studied species exhibit foliar, cauline, and root characters shared with other Cranichideae species (e.g., spiranthosomes, fiber rings, vascular bundles in the form of “^” or “v”, fleshy roots, among others). Others can be interpreted as adaptive strategies that have evolved in response to the environment and their terrestrial life form, including cuticle thickness, the presence of amphistomatic leaves, fibers, and lignin for internal tissues support, fleshy roots with reduced velamen compared to the cortex, the presence of trichomes to increase water and nutrients absorption, and raphides for calcium reserve, osmotic and ionic regulation, detoxification, and defense. In this study, the cataphylls are received in anatomical terms for Orchidaceae, in addition to verifying that the tiny starch grains are aggregated in spiranthosomes throughout the vegetative systems of *B. guayanensis*, and such structures, as well as the alkaloids, are first referred to leaves, especially for the stomatal guard cells. The presence of hyphae and pelotons in the stem of *B. guayanensis* are first described for Cranichideae. We believe that such fungal structures, located in the peripheral portions of the rhizomes, are important strategies for the maintenance of internal tissues and the efficient inoculation of still young adventitious roots. In addition, the presence and abundance of alkaloids and raphides in the reserve organs (roots and rhizomes), as well as in the guard cells of the stomata, may be associated with defense against herbivores and against the entry and systematization of pathogens and diseases, since its reserve organs are more durable than the aerial ones and are responsible for the maintenance and renewal of its aerial parts by storing water and other energy reserves (e.g., sugars and nutrients), and the stomata for configuring an entryway for harmful microorganisms. Additionally, *B. guayanensis* is likely to be a potentially medicinal species, mainly due to the abundance of alkaloids in its roots and rhizomes, similarly to phylogenetically related groups of the family. Furthermore, this study reveals the importance of anatomical and histochemical studies, in particular, directed to neotropical orchids that are poorly studied, guiding the development of studies with different aspects, especially biochemical and phytochemical studies.

## Data Availability

Data is contained within the article.
